# Sanitation for all: the global opportunity to increase transgenerational health gains and better understand the link between NCDs and NTDs, a scoping review

**DOI:** 10.1186/s40794-017-0051-3

**Published:** 2017-04-26

**Authors:** Shiva Raj Mishra, Meghnath Dhimal, Parash Mani Bhandari, Bipin Adhikari

**Affiliations:** 1 Nepal Development Society, P.O.Box. 75, Bharatpur-10, Nepal; 2 School of Population Health, University of Western Australia, Perth, WA 6009 Australia; 3 Nepal Health Research Council (NHRC), Ramshah Path, Kathmandu, Nepal; 4 Institute of Occupational Medicine, Social Medicine and Environmental Medicine, Goethe University, Frankfurt am Main, Germany; 5 Maharajgunj Medical Campus, Institute of Medicine, Kathmandu, Nepal; 6 Mahidol-Oxford Tropical Medicine Research Unit, Faculty of Tropical Medicine, Mahidol University, Bangkok, Thailand

**Keywords:** Neglected-tropical diseases, Non-communicable diseases, Chronic diseases, Asia, Africa, Sanitation

## Abstract

The global sanitation divide is narrowing. However, in many countries in Asia and Africa, the gap between rural and urban sanitation coverage is rather widening. Moreover, there is an increase in the burden of non-communicable diseases (NCDs), notwithstanding to the already high burden of neglected tropical diseases (NTDs). A scientific query is building on how the global ‘sanitation for all’ goal will address the dual burden of NTDs and NCDs, and help further understand the link between the two. This paper aims to discuss the link between i) sanitation and NTDs, and ii) sanitation and NCDs through a scoping review of the literature.

## Background

The global division in ‘sanitation for all’ is closing with studies showing 47% of people in South Asia and 30% of them in Sub-Saharan Africa in 2015 have achieved basic sanitation facilities compared to the baseline average of 22% and 24% respectively in 1990 [1]. Globally, this change equals to an increase in improved sanitation coverage from 54% in 1990 to 68% in 2015, and in the least developed countries, increase in sanitation coverage from 20% in 1990 to 38% in 2015 [1]. Despite this global progress, 950 million people, mostly those in low and middle-income countries (LMICs), defecated in the open in 2015.

Among the people without access to improved sanitation, seven of ten were rural inhabitants. In some parts of LMICs, the rural and urban sanitation gap is rather widening, with rural areas deprived of adequate sanitation facilities. In India, the number of people without improved sanitation has declined marginally (10% of urban inhabitants in 2015 compared to 29% in 1990 defecated in open areas while 61% rural inhabitants in 2015 compared to 91% in 1990 defecated in open areas) [2]. Due to inadequate sanitation, there is a wide spectrum of effects on maternal and child health, from water contaminations, resulting in diarrheal diseases including cholera and other gastrointestinal infections [3, 4]. While the spectrum of effects of inadequate sanitation so far has been established to affect the Neglected Tropical Diseases (NTDs) and communicable diseases, some studies have shown the link between inadequate sanitation and chronic non-communicable diseases (NCDs) through soil-transmitted helminths (STH) and other infections [5], and through other possible mechanisms. This observed link bridges the relationship between sanitation and NCDs and is discussed here in this article.

## Methods

The method chosen for this review uses the principles and practices of a scoping review [6, 7]. A scoping review principally includes the following five phases: i) formulating a research question, ii) identifying relevant studies, iii) selection of studies, iv) extracting and analysing results, and v) summarising and reporting the results. Using the several combinations of search terms: “sanitation”, “hygiene”, “WASH”, “non-communicable diseases”, “chronic diseases”, “neglected tropical diseases” and “low-income country”, literature search was carried out in PubMed/Medline and Google Scholar without limiting the search to language or study year. A total of 18 studies were obtained for Panel 1 (Review on the link between sanitation and NTDs), and 50 studies for Panel 2 (Review on the link between sanitation and NCDs). Further, we made an online post in ResearchGate (http://tinyurl.com/NCDsandNTDs) asking researchers to post published or unpublished studies, that they were aware of, on the link between NCDs and NTDs. Doing so, we identified ten additional papers which were later incorporated in Panel 2 of this paper.

## Main Text

### Inadequate Sanitation and Health

Some scholars like Sunita Narain correctly said that “rapidly-modernizing India is drowning in its own excreta” [8]. According to her estimates, little more than 33% of rural population in India compared to the 87% of city population have access to a toilet in India. However, having a toilet is not enough in itself. Inadequate management of excreta is another problem in many cities in India. This has been a common challenge in other LMICs as well.

The continuum of sewer management from having adequate sanitation facilities to adequate sewer treatment facilities is critical. Other researchers such as Abhishek Sharma advocate that economic benefits can be gained by improving sanitation coverage, asking for the commercialization of human excreta as a ‘business investment’ in Indian railways by building small biogas plants in trains or at stations [9]. The economic gains from having improved sanitation coverage have not been a topic of serious discussion among policy makers, and there has largely been a lack of interest in health leaders and health researchers to explore the health benefits of having and using toilets every day. However, the previous estimation states that every dollar spent in universal access to sanitation in countries not in Organisation for Economic Co-operation and Development will pay back 11.2 dollars [10].

In Panel 1, we summarise the link between NTDs and sanitation.

## Panel 1: Review on the link between sanitation and NTDs

NTDs are a group of chronic (disabling) diseases affecting more than a billion people residing in 149 countries worldwide, but most of these affect the poorest of the poor (bottom billion of the earth’s population) in regions with tropical and subtropical climates [11, 12]. It is estimated that for every dollar invested in NTD control, there will be an increase in economic productivity by 50 folds [13]. Often these diseases are called “neglected” because they are not prioritised in terms of control and funding as compared to the “big three” diseases - HIV/AIDS, malaria, and tuberculosis. However, in regards to mortality, one NTD alone kills more young children each year than all “big three” diseases combined [14].

The World Health Organization (WHO) has so far listed 17 diseases under its NTD programme: dengue fever, chikungunya fever, rabies, trachoma, buruli ulcer (i.e., *Mycobacterium ulcerans* infection), leprosy (*Mycobacterium leprae* infection), chagas disease (American trypanosomiasis), human African trypanosomiasis (sleeping sickness), leishmaniasis, cysticercosis, dracunculiasis (guinea-worm disease), echinococcosis, food-borne trematode infections, lymphatic filariasis, onchocerciasis (river blindness), schistosomiasis (bilharziasis), and soil-transmitted helminthiases [11].

According to the Global Burden of Disease Study 2010, the estimated disability-adjusted life years (DALYs) of these NTDs alone were 23.06 (95% CI: 20.30-35.12) million [15]. However, the real burden of NTDs in many regions and countries, especially in LMICs such as Nepal, is underreported. This is particularly due to the dysfunctional data collection system at the primary health care level and the unavailability of funding for research [16–18]. For the prevention of NTDs through sanitation, safe water, and hygiene (WASH) related programs, WHO has formulated a Global Strategy on ‘Water, Sanitation and Hygiene for accelerating and sustaining progress on Neglected Tropical Diseases’. A vicious circle of poverty and disease is contributed by NTDs which further adds burden to the already stretched health systems [19].

About one-third of the world’s population (2.4 billion) lacks access to adequate sanitation, one billion people practice open defecation, and 663 million do not have access to improved sources of drinking water [20]. Among the NTDs, schistosomiasis, trachoma and STH are strongly associated with WASH. The association of NTDs with WASH has been substantiated by some systematic reviews and meta-analyses [21–23]. Another study reports that better hygiene in children was associated with lower odds of trachomatous infection [22]. Similarly, WASH access and practice were associated with 33–70% lower odds of STH infection [23].

For prevention and treatment of many NTDs, WASH interventions play a critical role. For effective WASH interventions, there is an urgent need for inter-sectoral collaboration. WHO has recommended an integrated approach to overcome the global impact of NTDs through five interventions on the path to universal coverage: innovative and intensified disease management; preventive chemotherapy; studies on vector ecology and management; veterinary public health services; and the provision of safe WASH [11]. For instance, international efforts to eliminate trachoma as a blinding disease has been successfully implemented in many countries based on the WHO - developed SAFE strategy which stands for surgery for trichiasis, antibiotics, facial cleanliness and environmental improvement [24, 25].

Description of the role of WASH in schistosomiasis, trachoma and STH prevention and control is provided below:

- **Schistosomiasis** is caused by infection with parasitic blood flukes. People become infected when they come into contact with water bodies harbouring freshwater snails that have been infected with urine and faeces of infected people. The eggs of these parasites cause extensive damage to tissues and organs which can result in chronic infection and if untreated can lead to death. It is estimated that worldwide, nearly 250 million people are at risk of schistosomiasis [26]. The eggs of the parasites causing schistosomiasis are shed by infected individuals through the faeces and urine, which if deposited in surface water, contaminate the water and infect snails. People who are at high risk (those who bathe, wash clothes or work in the water) acquire larvae released from the snail which enters into the human skin [27]. Hence community-based sanitation facilities are essential to prevent contamination of water bodies with urine and faeces and infection of schistosomiasis.
- **Trachoma** is an infectious eye disease caused by bacterium *C. trachomatis* and is the leading cause of preventable blindness worldwide. Infection is caused by black flies that breed in human faeces and can contaminate humans by fingers, hands, clothing, or discharge from the eyes and nose of an infected individual. The population at risk of trachoma is estimated at 229 million [28]. Key hygiene activities to prevent trachoma include promoting regular face washing with soap to remove eye and nasal discharge contaminated with bacteria as well as promoting regular washing of clothing and bedding with soap.
  Sanitation interventions include encouraging communities to reduce open defecation, increasing access to and use of household toilets to minimise open defecation deposits near the home and promoting latrine maintenance. Improving access to clean water, which can lead to adequate water use for household hygiene practices (face washing and washing of clothing and bedding), can intercept the spread of disease [27].
- **Soil-transmitted helminths** refer to a group of parasites (roundworm, whipworm, and hookworm) that live in the soil in warm and humid climates, spread through physical contact with faeces of infected people, and survive in the human digestive system. It is estimated that 1 billion people are infected with STH or are at risk of infection worldwide. STH infection can cause blood loss leading to anaemia, nutritional deficiencies and thus is harmful in particular to children and women of childbearing age.
  It is estimated that over one billion people are at risk of infection with STH, including over 800 million children worldwide [26]. Key hygiene and sanitation interventions for prevention and control of STH include promoting hand washing before eating, after working, and after defecation; promoting proper disposal of infant/child feces; promoting wearing shoes when walking outside; reducing open defecation to minimize soil contamination; ensuring access to a household latrine and toilets in schools to minimize open defecation and; ensuring that processes are in place for regular cleaning and maintenance of toilets [27].

### Sanitation Links with NCDs

NCDs in the 21^st^ century are the leading killer diseases which contribute to 38 million deaths every year, and nearly three-quarter of these deaths occurs in LMICs. Also, nearly 42% of total NCD related deaths occur in people below the age of 70 [29]. Development of NCDs is multifactorial and is largely the result of lifestyle factors. Also, its link with NTDs is emerging as a new area for scientific enquiry. The early diagnosis of NTDs will have a dual advantage over late diagnosis and management. It can be a portal of entry for NCD screening and management because many of such NCDs are commonly prevalent among people with NTDs and are routinely diagnosed at primary health care level. Without further studies, we are not in the position to say comorbidities in NTDs are the result of NTDs because the pathological basis of NCDs manifestation in NTDs is miles away from the adequate explanation. NCDs are manifested in the cases of NTDs at the later stage of its prognosis, which may not be directly due to NTDs itself, but due to late outcomes of the diseases or the complications arising due to late health seeking behaviour.

In Panel 2, we present a summary of the link between NCDs and sanitation. Based on our cursory literature review, we also enlist some evidence on possible explanations for the links presented.

## Panel 2: Review on the link between sanitation and NCDs

### Cancer

The release of eggs into the fresh water bodies and consumption of raw fish are the two poor sanitary practices that are reported to facilitate the transmission of *Opisthorchis* species from their first intermediate host-snail to second intermediate host—fish and at the end to the final host—human [30]. *Opisthorchis* infestation is documented to be linked with cholangiocarcinoma [31, 32]. In experimental studies on the liver of golden hamsters, researchers observed the development of cholangiocarcinoma from the chronic infestation of *Opisthorchis* [33, 34]. However, controlled trials carried out among the human population are lacking.

In addition to *Opisthorchis* infestation, studies have also linked *Clonorchis* infestation with cholangiocarcinoma [32, 35–40]. Liver fluke induced chronic inflammation coupled with some specific microRNAs may explain the carcinogenesis from *Opisthorchis* or *Clonorchis* infection [41, 42].

Additionally, the chronic infection of schistosomiasis is documented to induce the metaplasia of transitional epithelial cells [43, 44] and to be a risk factor for the development of bladder cancer [45, 46]. T-helper 2 (Th2) type immune response instigated by *Schistosoma* or genetic polymorphisms mostly due to chronic infection may be a possible explanation for this carcinogenesis [47, 48].

Not only worms but also fungal species are reported to be associated with cancer. Chromoblastomycosis, a fungal infection more common in people with poor hygiene and inappropriate clothing or footwear [49], is observed to have an association with squamous cell carcinoma [50, 51]. However, the pathogenesis for the development of carcinoma from chromoblastomycosis is not well-understood.

### Diabetes

*Trypanosoma cruzi*, a parasite, is reported to enter from faeces of triatomine, an insect vector, into the human body through the mucous membrane or cracks in skin, and cause chagas disease. This illness is common among residents of areas with improper sanitation and poor housing [52]. The risk of development of diabetes is observed to be higher in women with chagas cardiomyopathy than those with chagas disease alone [53]. This may be due to the physiological and morphological changes in pancreas from chagas disease [54, 55].

Similarly, leprosy is transmitted via contact with nasal fluid infected with *Mycobacterium leprae* or *Mycobacterium lepromatosis* [56, 57] in the absence of adequate sanitation [58]. As reported by a study, in comparison to people without leprosy, the prevalence of diabetes is greater in people with such illness [59]. This may be due to high levels of pro-inflammatory cytokines such as TNFα [59] which induces insulin resistance in people with leprosy [60].

However, *Schistosoma* infection is reported to provide the protective effect against diabetes [61]. Similar to this, infestation of other soil-transmitted helminths, worms that harbour in filthy soils, are observed to have a protective effect against diabetes [62, 63]. This protective effect against diabetes may be due to activation of Th2 response, eosinophilia, and waist adipose tissue M2 polarisation from helminth infection [64, 65].

### Portal hypertension

Patients with schistosomiasis often develop portal hypertension [66]. This may be due to induction of osteopontin production by the eggs of *Schistosoma* which increases the severity of liver fibrosis [67].

### Cardiomyopathy

Development of cardiomyopathy is reported in the person with chagas disease [68]. Progression of chagas disease to cardiomyopathy is characterised by parasite-mediated injuries [69], immunological reactions [70], and microvascular damages [71].

In addition to this, serological tests have revealed the association of *Toxoplasma* infection with cardiomyopathy [72]. Macrophage production by cytokine activation [73] and production of IFN-γ and interleukin-12 [74, 75] are documented to be possible causes for the development of cardiomyopathy after *Toxoplasma* infection.

### Chronic kidney disease and renal failure

Chronic kidney disease, and often renal failure is observed in a person with schistosomiasis [76]. Schistosomiasis results in immune-complex mediated reactions [77] or activation of polyclonal B-lymphocytes [78], which may be responsible for the chronic kidney disease or renal failure.

### Asthma

Transmission of *Toxocora* from cats or dogs to human occurs from contact with their faeces. Lack of proper sanitation & hygiene makes a person susceptible to *Toxocora* infection [79], an infection which is associated with asthma [80]. This association may be due to the induction of Th2 immunity after a *Toxocora* infestation that triggers chronic allergic manifestations, finally leading to asthma [81].

Similarly, dust mites and respiratory infection by chlamydia or influenza virus, all of which groom in the absence of adequate hygiene, are also linked with chronic asthma [82, 83]. These links may be explained by the production of agent specific IgE after the infection [84, 85], which subsequently develops into a chronic asthma.

### Acute myocardial infarction

Acute respiratory infection, an infection that may be exacerbated by poor sanitation [86], is documented as a risk factor for acute myocardial infarction [87]. The risk of acute myocardial infarction from acute respiratory infection may be due to the increased concentration of C-reactive protein [88], or inflammatory cytokines [89], or platelet activation [90].

### Global strides in ‘sanitation for all’

A great stride has been achieved in ‘sanitation for all’ in recent years in LMICs despite public apathy to discuss or even talk about toilets. It is largely deliberated by the works of international organisations like United Nations Population Fund (UNFPA). For example, UNFPA has been conducting open defecation free campaigns in 30 districts of Nepal. From sanitation coverage of one percent, 25 years ago to 76% in 2014, Nepal has made a big stride in sanitation coverage. Fifteen districts, of Nepal’s 75 districts, were labelled open defecation free (ODF) in 2014, and another 15 are in the process of achieving that status [91]. Similar progress has been achieved in many other countries such as Bangladesh, Pakistan and India which have achieved a 30% or greater drop in population in ODF zones since the 1990s [1].

With the adoption of SDGs, the global goals have shifted from narrowly defined health goals to broader social goals. That includes ‘sanitation for all’ leading to sustainable development. Inadequate sanitation cannot be a choice, as it leads to social deprivation of rights and lack of access to toilets, which consequently increases the disease burden. SDGs should pivot the agenda of ‘sanitation for all’ to other SDGs and global goals on maternal and child health, and NCDs for the sustainable development and health of individual and society.

A greater involvement of civil society and the community is needed in both NTDs and NCDs related programs. Rather than a top-down project, a community-led project with a greater community ownership and engagement holds promise [92]. Securing the community’s initiative to plan, execute and supervise can be a more sustainable solution than vertical efforts of implementation where a community may feel obliged, marginalised or even neglected [92, 93]. Nonetheless, engaging communities for such projects requires a stepwise process of selecting the community leaders, training them, providing required resources, supervising them with additional training, resources and finally monitoring the activities with transparent performance data [93].

Finally, ‘sanitation for all’ appears to be not just the cure for many NTDs, but also many NCDs. However, existing evidence does not give us much confidence to discuss the benefits with certainty particularly on NCDs control and management. Future studies should explore dual benefits of sanitation coverage on NTDs and NCDs.

## Conclusions

This qualitative review is an attempt to document the health effects of inadequate sanitation from theoretical perspectives, with its link with NCDs and NTDs and global strides for ‘sanitation for all’. The current research gaps demands for more systematic reviews which can quantify health effects, economic effects and guide for the policy for the goal of sanitation. The global strides in ‘sanitation for all’ are welcoming, however, there are challenges to integrating sanitation with lifestyle in LMICs. In particular, the effect of inadequate sanitation and its effect in NCDs is poorly understood to this date. Without a doubt, investing in sanitation is a primary prevention for many diseases and conditions and is a great investment for mankind in the 21^st^ century. Greater investment with community engagement, strong commitment to global goals and newer strategies for ‘sanitation for all’ are urgently needed.

## Abbreviations

LMICs: Low and Middle-Income Countries
NCDs: Non-Communicable Diseases
NTDs: Neglected Tropical Diseases
SDGs: Sustainable Development Goals
WHO: World Health Organization
